# Assessing changes in adolescent girls’ and young women’s sexual and reproductive health service utilisation following a COVID-19 lockdown in eSwatini

**DOI:** 10.1080/16549716.2023.2243760

**Published:** 2023-08-11

**Authors:** Marie A. Brault, Erika L. Linnander, Thokozani M. Ginindza, Khabonina Mabuza, Sarah Christie, Maureen E. Canavan, Anastasia Jones, Mayur M. Desai

**Affiliations:** a Department of Health Promotion and Behavioral Sciences, University of Texas Health Science Center at Houston School of Public Health, San Antonio, TX, USA; b Department of Social and Behavioral Sciences, Yale School of Public Health, New Haven, CT, USA; c Global Health Leadership Initiative, Yale School of Public Health, New Haven, CT, USA; d Department of Health Policy & Management, Yale School of Public Health, New Haven, CT, USA; e Health Management Information Systems (HMIS), eSwatini Ministry of Health, Mbabane, eSwatini; f Project Last Mile, Mbabane, eSwatini; g Department of Epidemiology of Microbial Diseases, Yale School of Public Health, New Haven, CT, USA; h School of Public Health, University of the Western Cape, Bellville, Republic of South Africa; i Yale School of Medicine, Yale Cancer Outcomes, Public Policy and Effectiveness Research (COPPER) Center, New Haven, CT, USA; j Department of Epidemiology, Human Genetics & Environmental Sciences, School of Public Health, University of Texas Health Science Center at Houston, San Antonio, TX, USA; k Department of Chronic Disease Epidemiology, Yale School of Public Health, New Haven, CT, USA

**Keywords:** COVID-19, sexual and reproductive health, primary care utilization, adolescent girls and young women, eSwatini

## Abstract

The effects of COVID-19-associated restrictions on youth sexual and reproductive health (SRH) care during the pandemic remain unclear, particularly in sub-Saharan Africa. This study uses interrupted time series analyses to assess changes in SRH care utilisation (including visits for HIV testing and treatment, family planning, and antenatal care) adolescent girls’ and young women’s (AGYW; aged 15–24 years old) in eSwatini following COVID-19 lockdown beginning in March 2020. SRH utilisation data from 32 clinics in the Manzini region that remained open throughout the 2020 COVID-19 period were extracted from eSwatini’s electronic health record system. We tabulated and graphed monthly visits (both overall and by visit type) by AGYW during the two-year period between January 2019 and December 2020. Despite the March to September 2020 lockdown, we did not detect significant changes in monthly visit trends from 2019 to 2020. Our findings suggest little change to AGYW’s SRH utilisation in eSwatini during the 2020 COVID-19 lockdown period.

## Introduction

In the early months of the COVID-19 pandemic, researchers predicted that COVID-related lockdowns would cause disruptions to sexual and reproductive health (SRH) services, including human immunodeficiency virus (HIV) testing and treatment, family planning, and antenatal care, which are key elements of the Guttmacher-Lancet Commission’s essential package of SRH services [1–4]. In these studies, adolescent girls and young women (AGYW; aged 15–24 years old) in marginalised communities and low- and middle-income countries (LMICs) were expected to be disproportionately impacted by SRH service disruptions, due to pre-existing gender and power inequities limiting AGYW’s access and driving higher rates of SRH concerns compared to men [1,5–7].

Despite the growing literature documenting the impacts of COVID-19 on SRH and youth health care, findings remain mixed. Some studies identify substantial declines in SRH service utilisation in high-income countries (HICs) and LMICs, while other studies find modest disruptions [8–19]. There are fewer studies that disaggregate data by age to assess youth usage of SRH services, particularly in LMICs, with more focus on HICs or usage among all ages [9,11,13–15,17,20].

This paper uses data from eSwatini’s electronic health record to explore the extent to which a six-month COVID-19-associated lockdown may have affected AGYW’s SRH service utilisation in primary care clinics in eSwatini. A secondary objective of the study was to assess whether there were differential changes in AGYW’s SRH service utilisation at clinics that had piloted enhanced youth-centred services prior to COVID-19. Findings may be useful in understanding the resiliency of health systems facing potential disruptions in care.

## Methods

### Context

The Kingdom of eSwatini is home to approximately 1.3 million people and is divided into four regions that are further subdivided into 59 *tinkhundla* (municipalities). Over a third of the population is aged 10–24 years old. The current analysis builds on previous work that implemented and evaluated a strategic marketing campaign called Girl Champ [21–23], which provided enhanced youth-centred clinic programming and outreach to AGYW aged 15 to 24 years old at three clinics in the Manzini region. Both the parent study and the current analysis were approved by the Yale Institutional Review Board and the eSwatini Ministry of Health (MoH).

### Data collection and analysis

In this study, we examine trends in service utilisation at the three Girl Champ clinics and at other primary care clinics between January 2019 and December 2020, a 2-year period spanning a COVID-19-associated lockdown in March-September 2020. Health service utilisation data (numbers of visits by visit type) were extracted from the MoH’s Client Management Information System (CMIS) in March 2021. To be included in the analysis, clinics had to have been using the CMIS since 2018, and they had to continue providing primary care services throughout the 2020 COVID-19 period. Clinics were excluded if they were: a speciality clinic, closed, or transitioned to a COVID-specific facility. Of the 61 public clinics in Manzini that were using the CMIS as of 2020, 14 clinics were excluded because they were not using the CMIS until 2019 or later, and an additional 15 clinics were excluded based on other criteria noted above. The final sample of 32 clinics was distributed across urban, suburban, and rural catchment areas.

All of 2019 was included to account for seasonal variations potentially influencing utilisation, independent of COVID. In eSwatini, COVID stay-at-home orders were initiated on 27 March 2020, and lifted on 17 September 2020. We initially examined longitudinal trends in the average number of monthly clinic visits by AGYW for any reason, with attention to clinics exposed to the Girl Champ intervention prior to the onset of COVID-19 as well as clinics not exposed to Girl Champ (Figure S1). We then focused on visits for the following essential SRH services that were documented in the CMIS database: HIV testing, antiretroviral therapy (ART), family planning (visits to initiate and follow-up visits), and antenatal care (ANC; visits to initiate ANC and follow-up ANC visits) (Figure S2). Visits for specific services were not mutually exclusive, as a young woman may receive more than one service at a single visit.

We performed interrupted time series segmented regression analyses (ITSA) to test the effect of the COVID-19 lockdown on monthly clinic visits for any services as well as for specific SRH services using a single-group design [24]. We used an ordinary least squares regression model with Newey – West standard errors to account for autocorrelation and heteroscedasticity within our data, tested for serial autocorrelation, and adjusted for this serial autocorrelation, when necessary, by lagging of the data. The number of first family planning visits was the only visit type found to have an autocorrelation of errors and thus was lagged by one timepoint. All models included a time variable (month), a binary indicator for pre-post COVID-19 lockdown, and an interaction term between time and the COVID-19 lockdown variable. We graphed the actual and predicted average number of monthly visits and reported the time trends both prior to and following the COVID-19 lockdown, determining whether trends changed post-lockdown. To account for potential spurious findings due to multiple testing, we applied a Bonferroni correction to maintain an overall level of significance of 0.05 [25]. Our individual test alpha level for each comparison was 0.006.

## Results

### Trends in overall service utilization

**Figure 1.**
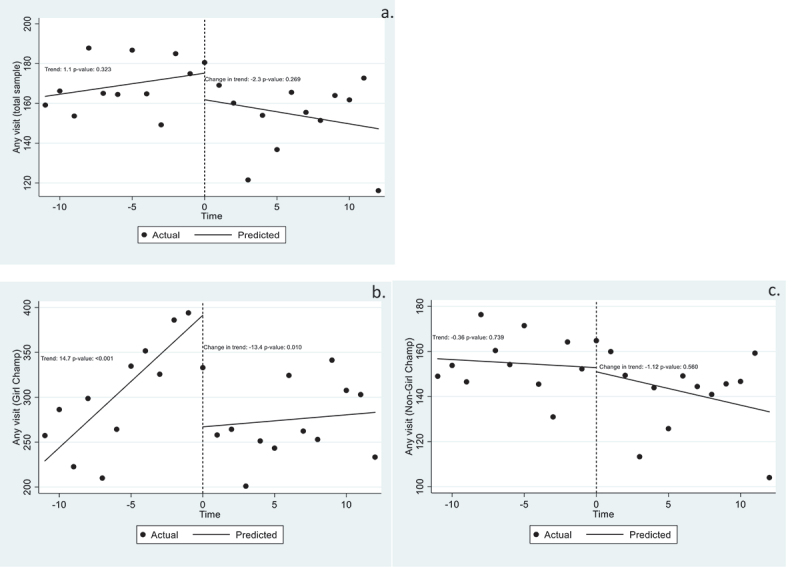
Average number of monthly clinic visits for all services by adolescent girls and young women. a. Clinic visits by AGYW across all sites. b. Clinic visits by AGYW at Girl Champ clinics. c. Clinic visits by AGYW at non-Girl Champ clinics. We conducted quintile comparisons across multiple visit types (9), and applied a Bonferroni correction to maintain an overall level of significance of 0.05. All individual tests required an alpha level of < 0.006 for statistical significance. See also Table S1.

Across all 32 clinics, there was no significant change after the COVID-19 lockdown in the monthly utilisation trend for all services (Figure 1, Panel a). This finding was consistent among the 29 non-Girl Champ clinics (Figure 1, Panel c). Among the three Girl Champ clinics (Figure 1, Panel b), there was a significant increasing trend in the average number of monthly visits for all services prior to the COVID-19 lockdown (trend of 14.7 additional visits per month [*p* < 0.001; 95% CI: 8.5, 21.0]). After the lockdown, we observed a decrease in the monthly trend of total visits to Girl Champ clinics, relative to the pre-lockdown period (13.4 fewer visits per clinic per month [*p* = 0.010; 95% CI: −23.2, −3.6]) (Figure 1, Panel b); however, this association was no longer statistically significant after applying the Bonferroni correction. Additionally, we did not detect longitudinal changes in monthly AGYW’s visits or visits to non-Girl Champ clinics from 2019 to 2020 (Figure 1, Panels a, c).

### Trends in SRH service utilization

**Figure 2.**
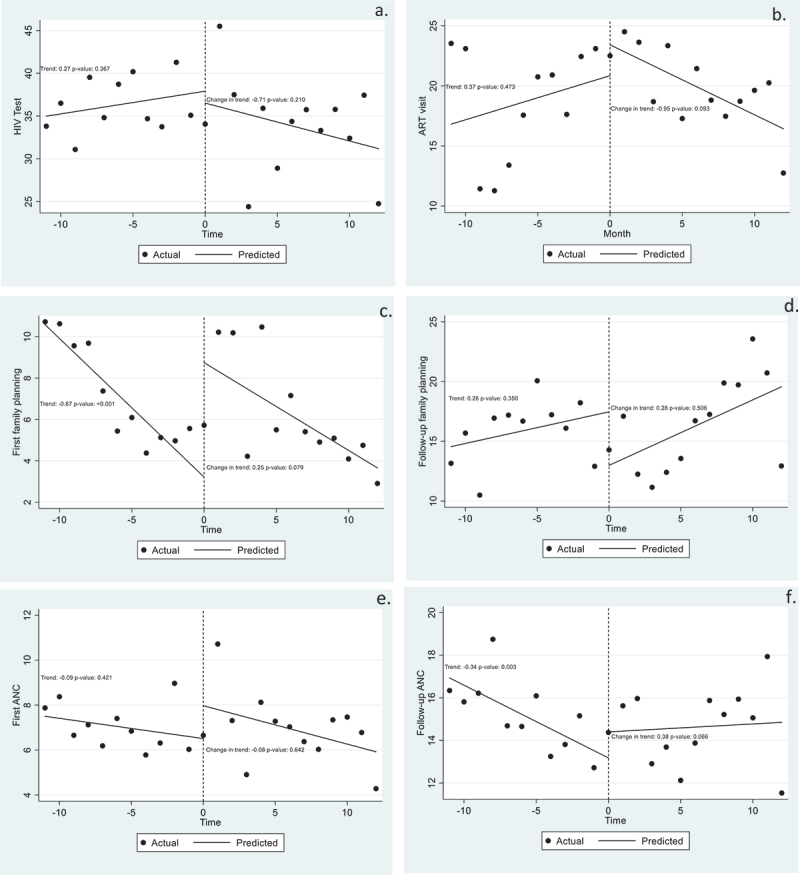
Panels a-f show monthly averages of adolescent girls’ and young women’s visits for specific sexual and reproductive health services. See also Table S1.

We also examined longitudinal change in utilisation for specific SRH services across all clinics (Figure 2, panels a-f). Prior to the COVID-19 lockdown, there were statistically significant decreasing trends in number of first family planning visits and of follow-up ANC visits; however, post-lockdown, no significant changes in trends were observed. Indeed, across each SRH service type, we did not detect COVID-related disruptions in service between 2019 and 2020.

## Discussion

Our results suggest there were no major interruptions in AGYW’s SRH service utilisation in the first 10 months of the COVID-19 pandemic. We note that what had been an increasing trend in overall service utilisation pre-lockdown among the three Girl Champ clinics changed in the post-lockdown period; however, this was not statistically significant after applying the Bonferroni correction, and no similar effect was found among the other 29 clinics included in the study. Our study adds to the findings concerning adolescent SRH service utilisation and access in LMICs during COVID-19, which demonstrate that some services and settings were more resilient to disruptions than others [11–14,16,17]. Additional qualitative and mixed methods research is needed to understand how clinics were able to maintain youth access to SRH services.

Our study should be interpreted in light of its limitations. First, our analyses focused on the first 10 months of the pandemic. While future trends in SRH service utilisation are not known, the study timeframe spanned a six-month COVID-19 lockdown, which was a period vulnerable to service disruption. Second, our sample of primary care clinics included those that stayed open to the general population throughout the study period. We do not quantify the loss of services in clinics that were transitioned to COVID-19 treatment facilities. In addition, we included clinics that were on the CMIS longer, and may have been more established organisations. We tried to limit potential bias by selecting a sample of clinics that ranged in size and geography, but recognise our sample may not be generalisable to all primary care centres in eSwatini. Finally, we acknowledge that there may have been other social and economic risk factors that influenced SRH service need and utilisation during COVID, such as increased rates of gender-based violence and unemployment [26–28]. However, the CMIS does not include such factors; thus, we cannot comment on what role these may have played. Despite the limitations, this study provides information for hypotheses that could be explored in future research. Further work with clinics that maintained SRH service access will be important in supporting health system resiliency in future health emergencies.

## Conclusions

Our findings suggest minimal changes in AGYW’s use of SRH services in eSwatini during the COVID-19 lockdown in 2020. Further studies should identify and disseminate strategies that clinics employed to sustain service delivery and AGYW’s engagement in care.

## Supplementary Material

Supplemental Material

## Paper context

Young women in Southern Africa face significant barriers to sexual and reproductive health (SRH) care access and use. It is unclear how COVID-19 lockdowns may have further influenced their use of care. We assessed adolescent girls’ and young women’s utilisation of SRH services in eSwatini before, during, and after COVID-19 lockdowns in 2020, and found that SRH utilisation remained consistent throughout. Future research should explore how clinics sustained service delivery during this time.
