# Supplementary material for: Assessing changes in adolescent girls’ and young women’s sexual and reproductive health service utilisation following a COVID-19 lockdown in eSwatini
Source: Glob Health Action. 2023 Aug 11;16(1):2243760. doi: 10.1080/16549716.2023.2243760 (PMC10424588; doi:10.1080/16549716.2023.2243760)
Supplement: Supplemental Material [file ZGHA_A_2243760_SM2449.docx]

**Supplementary Materials**


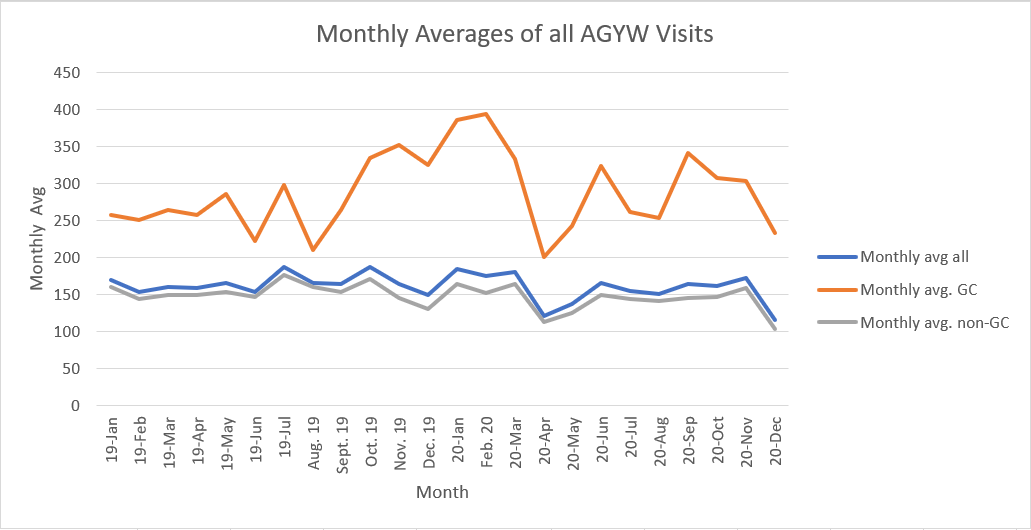


Initiation of Covid Restrictions

Covid Restrictions Lifted

Supplementary Figure 1: Monthly average of clinic visits by adolescent girls and young women.

B.

A.


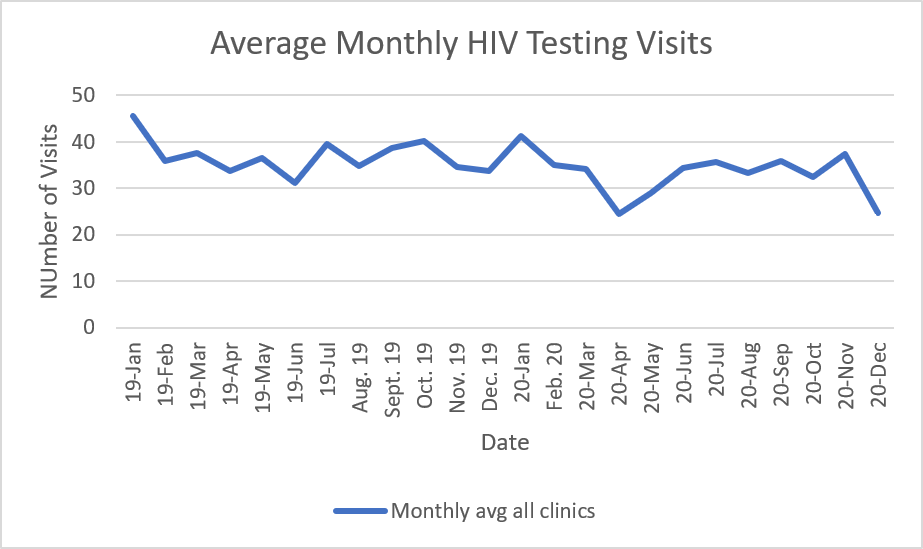


Initiation of Covid Restrictions

Covid Restrictions Lifted


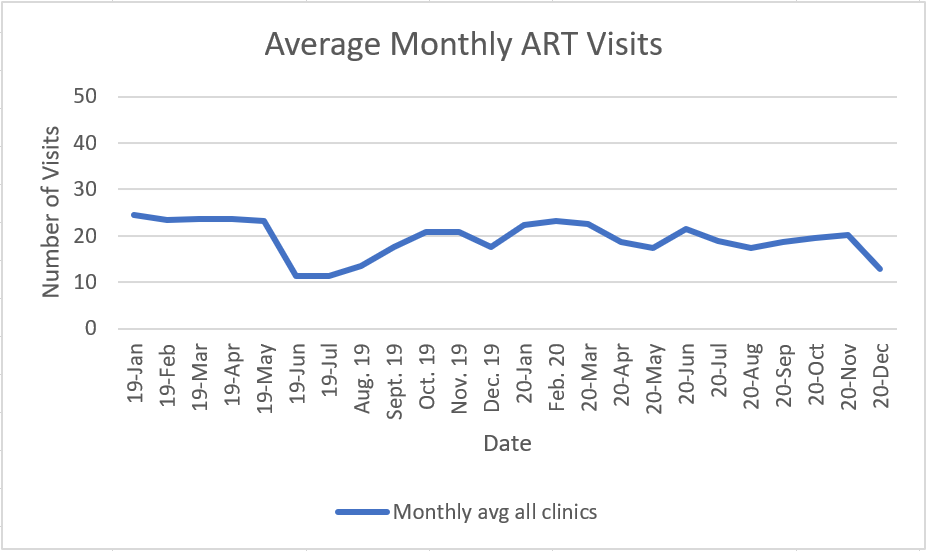


Initiation of Covid Restrictions

Covid Restrictions Lifted


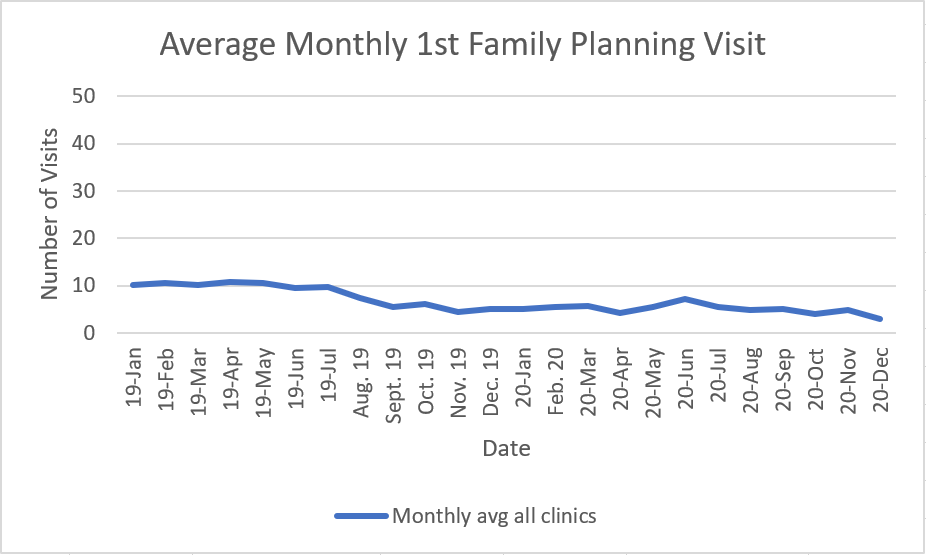


Initiation of Covid Restrictions

Covid Restrictions Lifted


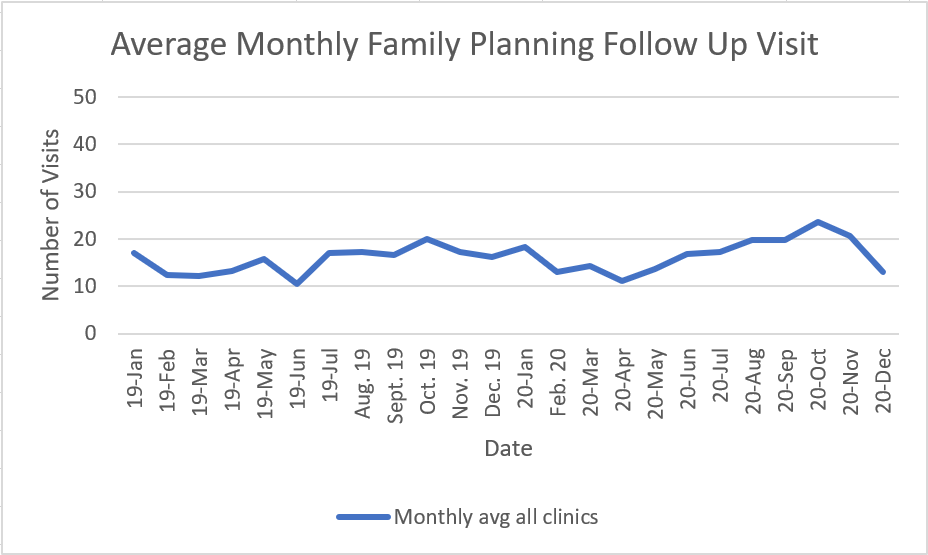


Initiation of Covid Restrictions

Covid Restrictions Lifted


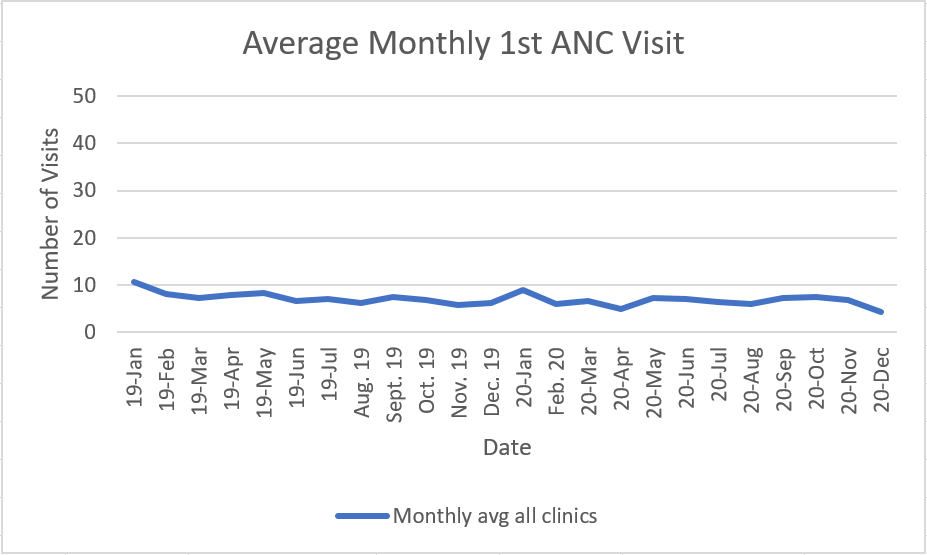


Initiation of Covid Restrictions

Covid Restrictions Lifted


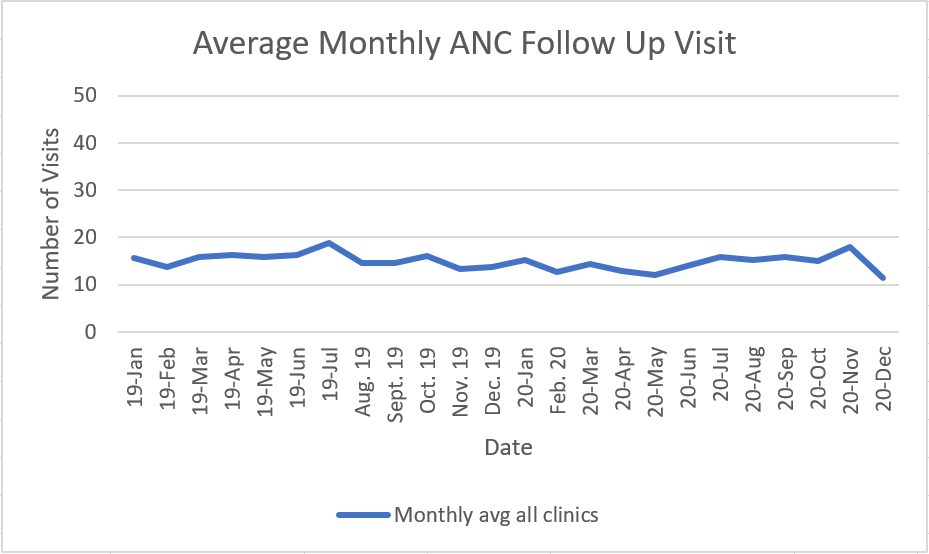


Initiation of Covid Restrictions

Covid Restrictions Lifted

D.

C.

F.

E.

Supplementary Figure 2: Panels A-F show monthly averages of adolescent girls’ and young women’s visits for specific SRH services.

**Supplementary Table 1**: Single group ITSA regression results for visits pre- and post- COVID lockdown.

|  | **Pre-lockdown trend** | **95% CI** | | ***p-value*** | **Post-lockdown trend^§^** | **95% CI** | | ***p-value*** |
| --- | --- | --- | --- | --- | --- | --- | --- | --- |
| Any visit (total sample) | 1.06 | -1.12 | 3.24 | 0.323 | -2.27 | -6.45 | 1.90 | 0.269 |
| Any visit (girl champ) | 14.74 | 8.45 | 21.04 | <0.001 | -13.40 | -23.19 | -3.61 | 0.010 |
| Any visit (non-girl champ) | -0.36 | -2.57 | 1.85 | 0.739 | -1.12 | -5.07 | 2.83 | 0.560 |
| HIV visit | 0.27 | -0.33 | 0.86 | 0.367 | -0.71 | -1.85 | 0.43 | 0.210 |
| ART visit | 0.37 | -0.68 | 1.41 | 0.473 | -0.95 | -2.07 | 0.17 | 0.093 |
| First family planning visit* | -0.67 | -0.90 | -0.44 | <0.001 | 0.25 | -0.03 | 0.52 | 0.079 |
| Follow-up family planning visit | 0.26 | -0.31 | 0.84 | 0.35 | 0.28 | -0.59 | 1.15 | 0.506 |
| First ANC visit | -0.09 | -0.32 | 0.14 | 0.421 | -0.08 | -0.44 | 0.28 | 0.642 |
| Follow-up ANC | -0.34 | -0.55 | -0.13 | 0.003 | 0.38 | -0.03 | 0.78 | 0.066 |
| **^§^** Relative to the pre COVID lockdown trend  *Autocorrelation addressed using Newey–West standard errors to calculate CI, with 1 lag. | | | | | | | | |
